# Hyperuricemia and clustering of cardiovascular risk factors in the Chinese adult population

**DOI:** 10.1038/s41598-017-05751-w

**Published:** 2017-07-14

**Authors:** Jie Wu, Ling Qiu, Xin-qi Cheng, Tao Xu, Wei Wu, Xue-jun Zeng, Yi-cong Ye, Xiu-zhi Guo, Qian Cheng, Qian Liu, Li Liu, Cheng-li Xu, Guang-jin Zhu

**Affiliations:** 1 Department of Clinical Laboratory, Peking Union Medical College Hospital, Peking Union Medical College & Chinese Academy of Medical Science, Beijing, 100730 China; 2 Department of Statistics, Institute of Basic Medical Sciences, Chinese Academy of Medical Sciences & Peking Union Medical College, Beijing, 100005 China; 3 Department of Internal Medicine, Peking Union Medical College Hospital, Peking Union Medical College & Chinese Academy of Medical Sciences, Beijing, 100730 China; 4 Department of Cardiology, Peking Union Medical College Hospital, Peking Union Medical College & Chinese Academy of Medical Sciences, Beijing, 100730 China; 5 Department of Pathophysiology, Institute of Basic Medical Sciences, Chinese Academy of Medical Sciences & Peking Union Medical College, Beijing, 100005 China

## Abstract

Hyperuricemia is common in China and the relevance of hyperuricemia and cardiovascular disease (CVD) risk has been highlighted, but to date there has been rarely nation-wide study in China. Here, we aim to estimate the current prevalence of hyperuricemia and evaluate the associations between hyperuricemia and cardiovascular risk factors (CRFs) clustering in a large sample of China adults including a plurality of ethnic minorities. Generally, a nationally representative sample of 22983 adults aged ≥18 years was recruited from 2007 to 2011. Questionnaire data and information on anthropometric characteristics, and laboratory measurements were collected. We define hyperuricemia as SUA ≥416 mmol/L for men and SUA ≥357 mmol/L for women. We found that the prevalence of hyperuricemia was 13.0% (18.5% in men and 8.0% in women). To our estimation, hyperuricemic subjects had higher prevalence rates of CRFs clustering than non-hyperuricemic subjects. Furthermore, there was a dose-response association between the number of CVD risk factors clustering and hyperuricemia. Our study revealed a high prevalence of hyperuricemia and CVD risk factors clustering among Chinese adults, and hyperuricemia was significantly associated with coexistence of more CVD risk factors. Therefore, guidance and effective lifestyle intervention are required to prevent hyperuricemia and CVD risk factors in China.

## Introduction

Cardiovascular disease (CVD) has become the leading cause of death in China and around the world^1, 2^. It has been known that dyslipidaemia, hypertension, diabetes, smoking and overweight/obesity are major risk factors for developing CVD^3, 4^. The clustering of these risk factors in the same individual will increase the incidence of future CVD significantly compared with a single risk factor^5^.

Hyperuricemia (HUA), which is caused by either overproduction or underexcretion of urate, has been always considered as the cause of gout due to accumulation of uric acid crystals^6, 7^. With the rapid economic growth and urbanization of China, the prevalence of hyperuricemia has significantly increased. Our previous study has shown that the prevalence of HUA is up to 13.7% in healthy adults from northern and northeastern Chinese provinces^8^. In recent years, dozens of surveys on HUA prevalence in mainland China have been conducted and a latest meta-analysis indicated that the pooled prevalence of hyperuricemia was 13.3% in Mainland China from 2000 to 2014^9^. As a large developing country, China has marked regional and ethnic differences, but to date, most studies on prevalence estimates for HUA have been limited to certain areas or nationality. Therefore, a nationally representative study on the epidemiology of hyperuricemia in China is needed.

A number of epidemiologic studies have confirmed an association between hyperuricemia and CVD^10, 11^. Elevated serum uric acid levels are strongly associated with cardiovascular risk^12, 13^. The relation between uric acid and cardiovascular risk is observed not only with hyperuricemia but also with uric acid levels in normal range^14^. In the last decades, several studies have assessed the role of SUA as a risk factor for CVD with conflicting results. A study in Japan has reported that hyperuricemia is positively associated with obesity, hypertension and dyslipidemia, and hyperuricemic subjects tend to have a clustering of these cardiovascular risk factors^15^. However, in the Chinese population, more studies have focused on the association of hyperuricemia with individual cardiovascular risk factors (CRFs) such as hypertension, diabetes, and correlation between hyperuricemia and clustering of multiple CRFs has been rarely reported.

Therefore, the objective of this study is to estimate the current prevalence of hyperuricemia and evaluate the association of hyperuricemia and CRFs clustering based on the recent, nationally representative samples of Chinese population.

## Results

### Prevalence of hyperuricemia

As shown in Table 1, the study included a total of 22983 people (10787 men and 12196 women), among whom 2977(13.0%) individuals were diagnosed as hyperuricemia. Men had a higher prevalence of HUA than women (18.5% vs. 8.0%, P < 0.001). The prevalence of hyperuricemia in men decreased with increasing age until 65 years, after which it increased progressively. In women, the prevalence of hyperuricemia increased in groups aged 35 years or older (P for trend <0.01). In both genders, the prevalence of hyperuricemia was higher in the southern and rural areas compared with northern and urban areas (all P < 0.01). Hui-Chinese had the lowest prevalence of hyperuricemia both in men (8.0%) and women (3.8%).

**Table 1:** Prevalence of hyperuricemia among study population.

	Men (n = 10787)	Women (n = 12196)	Total (n = 22983)
Overall	18.5 ± 0.37	8.0 ± 0.25	13.0 ± 0.22
Age group			
18–34yrs	19.1 ± 0.52	5.4 ± 0.55	12.1 ± 0.38
35–44yrs	19.1 ± 0.64	4.2 ± 0.62	10.8 ± 0.45
45–54yrs	16.7 ± 0.71	7.4 ± 0.67	11.6 ± 0.49
55–64yrs	16.4 ± 0.83	11.1 ± 0.78	13.5 ± 0.57
≥65yrs	21.2 ± 1.06	19.8 ± 1.11	20.5 ± 0.77
Regions			
South	19.9 ± 0.46	9.3 ± 0.46	14.4 ± 0.33
North	17.0 ± 0.43	6.7 ± 0.42	11.5 ± 0.30
Urban	16.4 ± 0.46	6.6 ± 0.46	11.2 ± 0.32
Rural	20.1 ± 0.43	9.0 ± 0.43	14.2 ± 0.30
Ethnic group			
Han	19.4 ± 0.40	9.3 ± 0.41	14.1 ± 0.29
Yi	15.7 ± 0.97	5.8 ± 0.92	10.2 ± 0.67
Hui	8.0 ± 0.75	3.8 ± 0.79	6.0 ± 0.54
Mongolian	19.5 ± 1.34	5.2 ± 1.20	11.0 ± 0.90
Korean	20.3 ± 1.72	8.6 ± 1.50	13.3 ± 1.13
Tibetan	23.0 ± 1.80	6.8 ± 1.65	13.4 ± 1.22
Tujia	21.2 ± 1.76	5.8 ± 2.14	14.3 ± 1.37
Miao	29.7 ± 2.43	5.8 ± 3.04	18.7 ± 1.92
Other	24.1 ± 2.67	7.2 ± 2.80	15.2 ± 1.94

Data are presented as percent prevalence ± standard error.

### The descriptive characteristics of the study population

The demographic and metabolic characteristics of the study participants are shown in Table 2. There was no significant difference in mean age between the normouricemic group and the hyperuricemic group for men, while the mean age of hyperuricemic group was significantly higher than normouricemic group for women. Regardless of gender, the hyperuricemic group, compared with the normouricemic group, had significantly higher values for body mass index (BMI), waist circumference (WC), systolic blood pressure (SBP), diastolic blood pressure (DBP), uric acid (UA), fasting blood glucose (FBG), triglyceride (TG), total cholesterol (TC), low density lipoprotein cholesterol (LDL-C), creatinine (Cr), blood urea nitrogen (BUN), but lower high-density lipoprotein cholesterol (HDL-C) levels and estimated glomerular filtration rate (eGFR) (all P < 0.001). Dyslipidemia, hypertension and overweight were more common in hyperuricemic subjects than in normouricemic subjects for both genders (P < 0.001).

**Table 2:** The characteristics of the study participants.

	Men (n = 10787)		Women (n = 12196)		*P-value* ^a^	*P-value* ^b^
	Non-HUA (n = 8788)	HUA (n = 1999)	Non-HUA (n = 11218)	HUA (n = 978)		
Age (years)	43.0 (29.3–56.8)	42.0 (29.8–57.6)	42.7 (31.0–55.0)	54.3 (38.0–65.7)	0.969	<0.001
BMI (kg/m^2^)	23.6 ± 3.43	25.3 ± 3.63	23.1 ± 3.33	25.0 ± 3.68	<0.001	<0.001
WC (cm)	81.4 ± 10.1	86.5 ± 10.6	76.1 ± 9.5	83.3 ± 10.5	<0.001	<0.001
SBP (mmHg)	127.6 ± 17.1	130.2 ± 17.4	122.7 ± 18.8	132.6 ± 22.8	<0.001	<0.001
DBP (mmHg)	79.8 ± 11.3	81.8 ± 11.7	77.7 ± 10.9	81.3 ± 11.9	<0.001	<0.001
UA (μmol/L)	317.2 ± 59.4	473.2 ± 53.3	246.1 ± 52.8	401.2 ± 46.6	<0.001	<0.001
FBG (mmol/L)	5.40 ± 1.24	5.54 ± 1.16	5.30 ± 1.06	5.72 ± 1.29	<0.001	<0.001
TG (mmol/L)	1.22 (0.84–1.85)	1.76 (1.05–2.84)	1.09 (0.77–1.59)	1.62 (1.11–2.52)	<0.001	<0.001
TC (mmol/L)	4.60 ± 1.04	4.88 ± 1.13	4.70 ± 1.01	5.11 ± 1.16	<0.001	<0.001
HDL-C (mmol/L)	1.31 ± 0.34	1.25 ± 0.32	1.47 ± 0.34	1.37 ± 0.33	<0.001	<0.001
LDL-C (mmol/L)	2.68 ± 0.84	2.78 ± 0.90	2.66 ± 0.85	2.95 ± 0.95	<0.001	<0.001
Cr (mg/dL)	81.0 ± 14.3	87.9 ± 22.8	63.1 ± 13.6	71.5 ± 19.4	<0.001	<0.001
BUN (mg/dL)	5.20 ± 1.42	5.45 ± 1.62	4.69 ± 1.38	5.44 ± 1.63	<0.001	<0.001
eGFR (mL/min/1.73 m2)	99.5 ± 18.3	94.0 ± 20.8	101 ± 19.3	87.3 ± 22.3	<0.001	<0.001
Dyslipidemia	4549 (51.8%)	1350 (67.5%)	4776 (42.6%)	655 (67.0%)	<0.001	<0.001
Hypertension	2285 (26.0%)	656 (32.8%)	2271 (20.2%)	366 (37.4%)	<0.001	<0.001
Diabetes	431 (4.9%)	102 (5.1%)	356 (3.2%)	96 (9.8%)	0.735	<0.001
Current smoking	3788 (43.1%)	838 (41.9%)	314 (2.8%)	37 (3.8%)	0.337	0.081
Overweight	2906 (33.1%)	1065 (53.3%)	2930 (26.1%)	457 (46.7%)	<0.001	<0.001

Data are presented as mean ± standard deviation (SD), n (%); Age and TG were reported as medians (interquartile range). HUA, hyperuricemia; BMI, body mass index; WC, waist circumference; SBP, systolic blood pressure; DBP, diastolic blood pressure; UA, uric acid; FBG, fasting blood glucose; TG, triglyceride; TC, total cholesterol; HDL-C, high density lipoprotein cholesterol; LDL-C, low density lipoprotein cholesterol; Cr, creatinine; BUN, blood urea nitrogen; eGFR, estimated glomerular filtration rate. ^a^Non-HUA vs. HUA in men; ^b^Non-HUA vs. HUA in women.

### Prevalence of CRFs clustering

**Figure 1:**
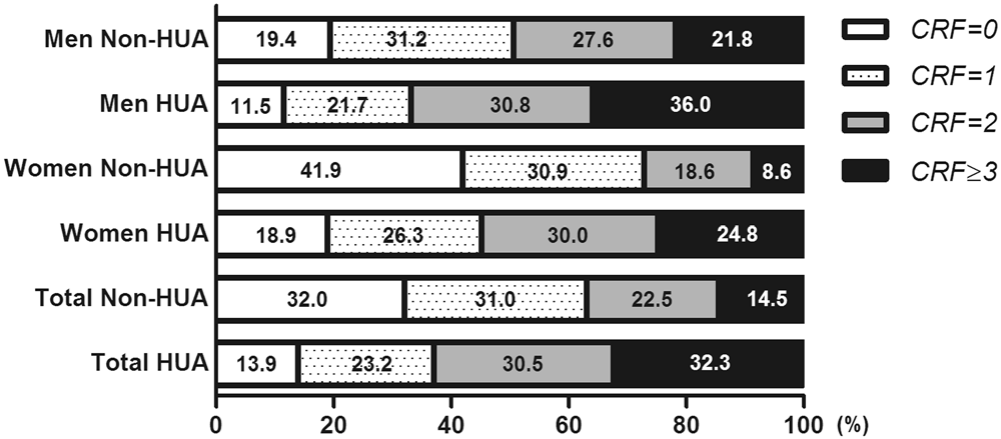
Prevalence of CRFs clustering among normouricemic and hyperuricemic adults. HUA, hyperuricemia; CRFs, cardiovascular risk factors, including dyslipidemia, hypertension, diabetes, current smoking, and overweight.

Figure 1 showed the clustering of major cardiovascular risk factors (CRFs), including dyslipidemia, hypertension, diabetes, current smoking and overweight, in normouricemic and hyperuricemic groups. 32%(men 19.4% and women 41.9%) of normouricemic adults, and 13.9%(men 11.5% and women 18.9%) of hyperuricemic adults had no major CRFs. In contrast, 31.0%, 22.5% and 14.5% of normouricemic adults, and 23.2%, 30.5% and 32.3% of hyperuricemic adults had 1, 2 and ≥3 CRFs, respectively. Normouricemic subjects were more likely to have 0 and 1 CRF than hyperuricemic subjects in both genders (all P < 0.01), while hyperuricemic subjects were more likely to have 2 and ≥3 CRFs than normouricemic subjects (all P < 0.01).

The age-stratified prevalence of clustered CRFs among normouricemic and hyperuricemic participants was shown in Table 3. The prevalence of ≥1, ≥2 and ≥3 CRFs was significantly higher in hyperuricemic men (88.5%, 66.8% and 36.0%) and hyperuricemic women (81.1%, 54.8% and 24.8%) than in normouricemic men (80.6%, 49.4% and 21.8%) and normouricemic women (58.1%, 27.2% and 8.6%) respectively. Moreover, for different age groups, the prevalence of ≥1, ≥2 and ≥3 CRFs in hyperuricemic group was all higher than in normouricemic group in both genders. In women, the prevalence of ≥1, ≥2 or ≥3 risk factors was increased with age. However, for men, the prevalence of ≥1, ≥2 or ≥3 risk factors increased progressively with increasing age until 55 years old, then decreased slightly.

**Table 3:** Prevalence of clustered CRFs among participants by gender and age.

clustered CRFs	Men			Women		
	Non-HUA	HUA	*P-value*	Non-HUA	HUA	*P-value*
≥1 risk factors						
Overall	7080 (80.6)	1770 (88.5)	<0.001	6517 (58.1)	793 (81.1)	<0.001
Age, y						
18–34	1907 (64.4)	536 (76.8)	<0.001	968 (27.0)	94 (46.3)	<0.001
35–44	1507 (87.7)	387 (95.6)	<0.001	1400 (54.1)	85 (74.6)	<0.001
45–54	1459 (90.6)	314 (96.9)	<0.001	1695 (76.7)	162 (91.5)	<0.001
55–64	1222 (89.3)	257 (95.9)	<0.001	1534 (86.6)	208 (93.7)	0.001
≥65	985 (87.2)	276 (90.8)	0.01	920 (86.6)	244 (93.1)	0.002
≥2 risk factors						
Overall	4339 (49.4)	1336 (66.8)	<0.001	3049 (27.2)	536 (54.8)	<0.001
Age, y						
18–34	821 (27.7)	318 (45.6)	<0.001	177 (4.9)	39 (19.2)	<0.001
35–44	984 (57.2)	333 (82.2)	<0.001	534 (20.6)	42 (36.8)	<0.001
45–54	1031 (64.0)	268 (82.7)	<0.001	853 (38.6)	108 (61.0)	<0.001
55–64	855 (62.5)	203 (75.7)	<0.001	908 (51.3)	161 (72.5)	<0.001
≥65	648 (57.3)	214 (70.4)	<0.001	577 (54.3)	186 (71.0)	<0.001
≥3 risk factors						
Overall	1913 (21.8)	720 (36.0)	<0.001	969 (8.6)	243 (24.8)	<0.001
Age, y						
18–34	279 (9.4)	155 (22.2)	<0.001	20 (0.6)	10 (4.9)	<0.001
35–44	448 (26.1)	181 (44.7)	<0.001	135 (5.2)	16 (14.0)	<0.001
45–54	506 (31.4)	161 (49.7)	<0.001	254 (11.5)	40 (22.6)	<0.001
55–64	404 (29.5)	110 (41.0)	<0.001	331 (18.7)	77 (34.7)	<0.001
≥65	276 (24.4)	113 (37.2)	<0.001	229 (21.6)	100 (38.2)	<0.001

Data are presented as N(percent prevalence). HUA, hyperuricemia; CRFs, cardiovascular risk factors.

### Multivariable Logistic Regression Analysis

Table 4 showed the results of multivariable logistic regression analysis for hyperuricemia. Except for current smoking, dyslipidemia, hypertension and overweight were statistically significant risk factors and positively correlated with hyperuricemia in both genders. However, diabetes was positively correlated with hyperuricemia only in women. We further analyzed the association between CVD risk factors clustering and hyperuricemia. As shown in Table 5, the results of multivariable logistic analysis revealed that the CVD risk factors clustering was significantly and positively associated with hyperuricemia when the 0 CVD risk factors were set as the reference category. After adjustment for age and ethnicity, the individuals with clustering of 1, 2 or ≥3 CVD risk factors were still more likely to have hyperuricemia compared with those without risk factors in both genders. CVD risk factors clustering were associated with the higher ORs of hyperurciemia in women than in men. Moreover, there was a dose-response association between the number of CVD risk factors clustering and hyperuricemia in both genders (P trend <0.001). A stronger association of hyperuricemia with clustered CVD risk factors was found in females than in males.

**Table 4:** OR and 95%CI of hyperuricemia associated with major CVD risk factors.

CVD risk factors	Men		Women	
	Unadjusted OR (95%CI)	Adjusted OR (95%CI)	Unadjusted OR (95%CI)	Adjusted OR (95%CI)
Dyslipidemia	1.94 (1.75–2.15)*	1.58 (1.41–1.76)*	2.74 (2.38–3.14)*	1.70 (1.46–1.98)*
Hypertension	1.39 (1.25–1.54)*	1.15 (1.02–1.29)*	2.36 (2.05–2.70)*	1.37 (1.18–1.60)*
Diabetes	1.04 (0.84–1.30)	0.82 (0.65–1.03)	3.32 (2.62–4.20)*	1.82 (1.42–2.34)*
Current smoking	0.95 (0.86–1.05)	0.94 (0.85–1.04)	1.37 (0.96–1.95)	1.11 (0.78–1.60)
Overweight	2.31 (2.09–2.55)*	2.03 (1.82–2.25)*	2.48 (2.17–2.83)*	1.82 (1.42–2.34)*

Age, ethnicity and all other risk factors were adjusted when estimate odds ratios (ORs) with 95% confidence intervals (CIs) of each variable. *p < 0.01.

**Table 5:** OR and 95%CI of hyperuricemia associated with clustered risk factors.

clustered CVD risk factors	Men		Women	
	Unadjusted OR (95%CI)	Adjusted OR (95%CI)	Unadjusted OR (95%CI)	Adjusted OR (95%CI)
0	1.00 (ref)	1.00 (ref)	1.00 (ref)	1.00 (ref)
1	1.18 (1.00–1.40)	1.22 (1.03–1.45)	1.88 (1.55–2.29)	1.49 (1.22–1.83)
2	1.89 (1.61–2.23)	2.03 (1.72–2.40)	3.58 (2.96–4.33)	2.49 (2.01–3.07)
≥3	2.81 (2.39–3.30)	3.04 (2.57–3.59)	6.37 (5.20–7.81)	4.06 (3.23–5.11)
P trend	<0.001	<0.001	<0.001	<0.001

Age and ethnicity were adjusted when estimate odds ratios (ORs) with 95% confidence intervals (CIs).

## Discussion

Our study revealed a high prevalence of hyperuricemia and demonstrated that hyperuricemia was associated with clustering of major CVD risk factors in the Chinese adult population. To our knowledge, this is the first multicenter study to analyze the association between hyperuricemia and clustering of multiple CVD risk factors in diverse Chinese provinces, with a large and representative study population including a plurality of ethnic minorities.

We observed that the prevalence of hyperuricemia correlated negatively with age in men aged <65 years, while in women aged 35 years or older, the prevalence of hyperuricemia correlated positively with age. The gender-related differences in the correlation between hyperuricemia and age has also been found in several other studies^15–17^, which may be because of the interactions of sex hormones^18, 19^. Although several previous studies have shown that the prevalence of hyperuricemia is higher in urban populations^9, 20^, the data in this study showed that HUA were more common among people living in rural compared with urban areas. Lifestyle, food, and race differences in China may affect the prevalence of hyperuricemia between areas.

It has been shown that hyperglycemia increases uric acid excretion, possibly by impairing tubular reabsorption of uric acid. In present study, we found that diabetes was more common in hyperuricemic women compared to normouricemic women. A possible explanation may be that diabetes patients in this study are in relatively mild condition, not enough to cause renal tubular reabsorption damage. In addition, some medication for hypertension like thiazide increase serum uric acid levels, which may be another possible reason.

Elevation of the SUA level has been known associated with major CRFs, such as hypertension, insulin resistance, dyslipidemia and obesity, which are hallmarks of metabolic syndrome^21–24^. Similar to other studies, in this study, individuals with hyperuricemia had significantly higher BMI, WC, BP, FBG, TG, TC, and LDL-C, but lower HDL-C, and higher prevalence of major CRFs, including dyslipidemia, hypertension and overweight. Interestingly, in women, there was a significantly association between hyperuricemia and diabetes, but in men no significantly association was found, which is likely due to differences in occupations, family responsibilities and health concerns. Prior cohort studies also found gender-specific differences regarding the relationship between uric acid levels and diabetes^25, 26^, further supporting our conclusion. Our study also indicated that individuals with hyperuricemia had significantly worse renal function. Considering that hyperuricemia has been implicated as a risk factor for renal disease^27–29^, further longitudinal studies are needed to clarify the causality.

Many published studies have demonstrated that CVD incidence and all-cause mortality increased markedly in the presence of CRFs clustering^30, 31^. Our findings showed that participants with hyperuricemia were more likely to have clustered CRFs than those without hyperuricemia, which suggest an association between hyperuricemia and an increased rate of cardiovascular events and higher mortality^32, 33^. Furthermore, we found that there was a dose-response association between the number of CRFs clustering and hyperuricemia. The greater number of CRFs clustering was associated with the higher odd ratio for hyperuricemia in both genders. However, a stronger association of hyperuricemia with CVD risk factors clustering was found in females than in males. Consistent with our results, a recent large study in Chinese showed that SUA and MS were much more closely related in females than in males^34^. In fact, several recent studies have evaluated gender-related differences in the association between hyperuricemia and cardiovascular events and obtained similar results. Ndrepepa *et al*. showed a strong association between hyperuricemia and an increased risk of mortality in both genders, with a stronger association in women^35^. A latest meta-analysis indicated that hyperuricemia may increase the risk of CVD, particularly CVD mortality in females^36^. Moreover, some researchers have shown that the significant association between high uric acid levels and CVD was found only in woman^37, 38^. Differences in cardiovascular risk profile may explain the stronger association between hyperuricaemia and cardiovascular events in women.

The strengths of the present study are that it is a population-based study with a representative sample of the general Chinese adult population, and the large sample size ensures sufficient power in estimating the prevalence of hyperuricemia and its association with CRFs clustering. In addition, the standardized testing process was applied in this multi-central study and all biochemical laboratories participating in the survey followed the same internal quality control program, which ensure the reliability and comparability of test results.

The present study has several limitations, however. Firstly, this is a cross sectional study, the relationship between hyperuricemia and CRFs clustering found in this paper could not be a considered established causality. Secondly, the major CRFs including dyslipidemia, diabetes, and hypertension were assessed mainly based on a single measurement of the corresponding parameters according to the commonly used international standards and further follow-up was not conducted, which maybe affected the accuracy of diagnosis of the major CRFs. Thirdly, reporting bias could not be avoided because many factors were self-reported, such as smoking, drugs. And certain drugs maybe affect serum uric acid levels, but we have no detailed data reported by subjects on these drugs usage in this study. For example, fenofibrate, an antilipotropic drug, decreases serum uric acid by increasing urinary excretion^39^. Thiazide, a diuretics drug, increases serum uric acid by stimulating urate reabsorption in the proximal tubule^40^. Taken together, compared to a cohort study, there are some common limitations that cross-sectional studies have in this study, which may lead to results bias. However, the relationship between hyperuricemia and CRFs clustering found in this study is clear and these limitations will not affect this conclusion.

In conclusion, the prevalence of hyperuricemia and CVD risk factors clustering is high among Chinese adults and hyperuricemia was significantly associated with CVD risk factors clustering. It appears that a relationship exists between uric acid and CVD, particularly in subjects at high risk for cardiovascular disease and in women, so further studies will be helpful to evaluate the effects of SUA lowering therapies on CVD prevention and outcome, especially in women.

## Methods

### Study population

**Figure 2:**
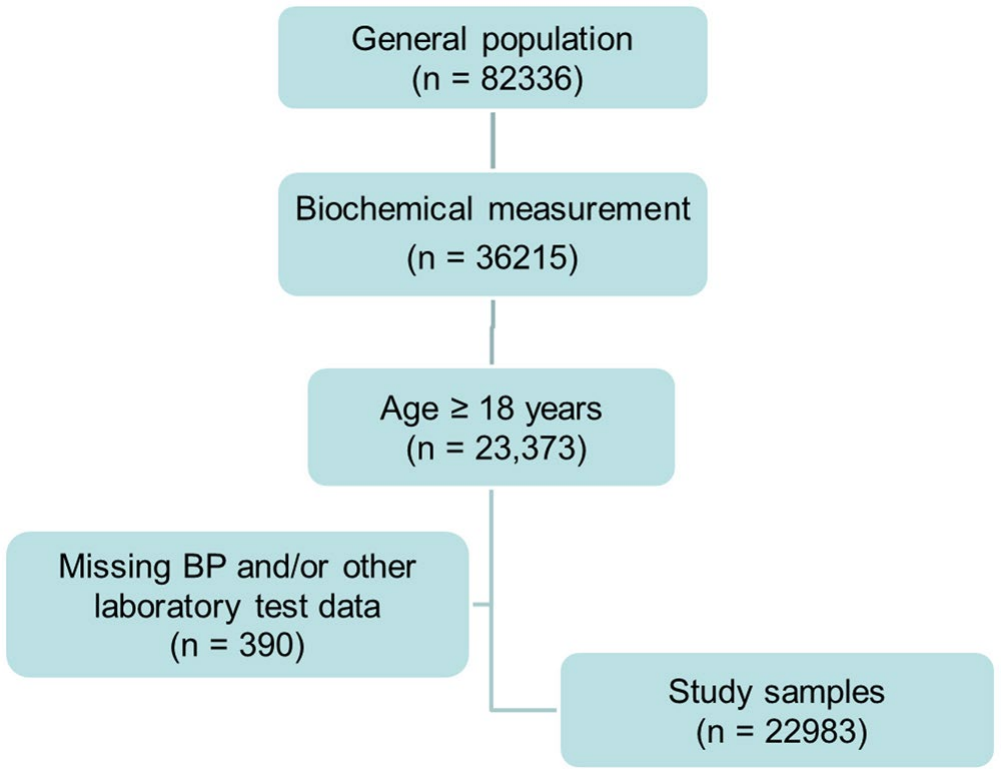
A schematic used for screening and inclusion of the study sample. A total of 82336 apparently healthy individuals were recruited between 2007 and 2011, and 36215 individuals had biochemistry measurements collected. Of the 23373 adults aged ≥18 years, 390 participants had missing data on BP and/or laboratory tests, and the final sample size was 22983.

Data used in the current analysis were derived from the Chinese Physiological Constant and Health Condition (CPCHC) survey, a population-based, cross-sectional survey, which was carried out from 2007 to 2011^41^. In this survey, a random, multistage, stratified sampling method was used to obtain a nationally representative sample of the general Chinese population. In brief, 6 provinces located in different geographical regions of China were selected and then 2 or 3 cities were sampled based on their economic status and the presence of ethnic minorities. Last, several communities were randomly selected from each city. In these selected communities, all eligible people were referred to as our survey subjects. Eligible people included 82336 apparently healthy participants aged 10–80 years old who weren’t suffering from severe systemic diseases (such as cardiovascular, renal, gastro-intestinal, pulmonary disease or cancer) and had not run a high fever in the past 15 days. After signing informed consent forms, all subjects came to the temporary physical examination centers voluntarily to take part in the survey. A schematic of the screening process is presented in Fig. 2. Among 82336 eligible subjects, 36215 people (44.0%) were selected randomly to complete blood biochemical testing. Of the 36215 participants, only subjects (n = 23373) aged ≥18 years were selected in the current analyses. Of these, a subset of 390 had incomplete information or missing data for blood pressure (BP) and/or blood sample parameters. Therefore, the final 22983 people (10787 men and 12196 women) were eligible to be included in the analysis.

The study was conducted in accordance with the Helsinki Declaration and was approved by the Ethics Committee of the Institute of Basic Medical Sciences at the Chinese Academy of Medical Sciences. Written informed consent was obtained from each participant before data collection.

### Data collection and anthropometry

Data was collected on all subjects via a standard questionnaire, which included demographic data, medical history, family history, smoking and alcohol drinking status. In order to ensure the compliance of the subjects and the truthiness of survey, we carried out health education for the subjects in advance by issuing leaflets and other forms, making them familiar with the research objectives and the precautions. Weight was measured to an accuracy of 0.1 kg and height was accurate to 0.1 cm, and body mass index (BMI) was calculated as weight in kilograms divided by the square of the height in meters. Waist circumference (WC) was measured with flexible and inelastic tape at the end of a gentle expiration. After a resting period of at least 10 minutes, blood pressure (BP) was measured three times using an Omron HEM-7000 electronic sphygmomanometer (Omron Healthcare; Muko, Kyoto, Japan) and the averages of the three measurements were used.

The staff who participated in the project, including doctors, medical students, nurses and medical technicians, was trained to ensure the consistency and accuracy of the operation. Specifically speaking, trained doctors and medical students are mainly responsible for the questionnaire, blood pressure measurement and physical examination, nurses responsible for blood collection, and medical technicians responsible for blood test. Finally, researchers, physicians, and statistical professionals analyze all the data and determine the major CRFs based on diagnostic criteria and test indicators.

### Laboratory measurements

In the morning overnight fasting blood was drawn by venipuncture. The blood specimens were centrifuged at 3000 rpm for 10 minutes and the serum was separated, and then sent to the laboratory where it was stored at −80 °C until the laboratory assays were performed. Serum total cholesterol (TC), triglycerides (TG), high-density lipoprotein cholesterol (HDL-C) and low-density lipoprotein cholesterol (LDL-C) were measured using the Beckman AU Series Automatic Biochemical Analyzer and Sekisui Medical (Japan) reagents. Uric acid (UA) and fasting blood glucose (FBG) levels were measured using the same instrument and Beckman AU reagents. The biochemical laboratories participating in the survey followed the common internal quality control program, which was standardized by the Peking Union Medical College Hospital.

### Diagnostic criteria for hyperuricemia and the major CRFs

Hyperuricemia was defined as SUA ≥7 mg/dl (416 mmol/L, male) or SUA ≥6 mg/dl (357 mmol/L, female), which is a widely accepted diagnostic criteria^42^. Dyslipidemia was defined as having at least one of the following: TC ≥5.2 mmol/L, TG ≥1.7 mmol/L, HDL-C <1.0 mmol/L, LDL-C ≥3.4 mmol/L. Hypertension was defined as having an average systolic blood pressure (SBP) ≥140 mm Hg and/or diastolic blood pressure (DBP) ≥90 mm Hg. Diabetes was defined as FBG ≥7.0 mmol/L and/or current antidiabetes medication use. Overweight/obesity was defined as having a BMI ≥25 kg/m^2^. Current smoking was classified as self-reported responses of “yes” to the question “Do you smoke cigarettes now?”

### Statistical analysis

Data were analyzed using SPSS 16.0 software (SPSS Inc., Chicago, IL, USA). Normally distributed continuous variables are presented as means ± standard deviation (SD), and were analyzed by t-test. Skewed distributed variable TG is presented as medians (interquartile range) and were compared by the Wilcoxon rank sum test. Categorical data are presented as percentages and were compared by the Chi-squared test. Odds ratios (ORs) and 95% confidence intervals (CI) were calculated by multivariable logistic regression to estimate the association between hyperuricemia and CVD risk factors. Two-tailed p < 0.05 was defined as statistically significant.
